# Supplementary figures and images for: Early administration of tecovirimat shortens the time to mpox clearance in a model of human infection
Source: PLoS Biol. 2023 Dec 21;21(12):e3002249. doi: 10.1371/journal.pbio.3002249 (PMC10734935; doi:10.1371/journal.pbio.3002249)

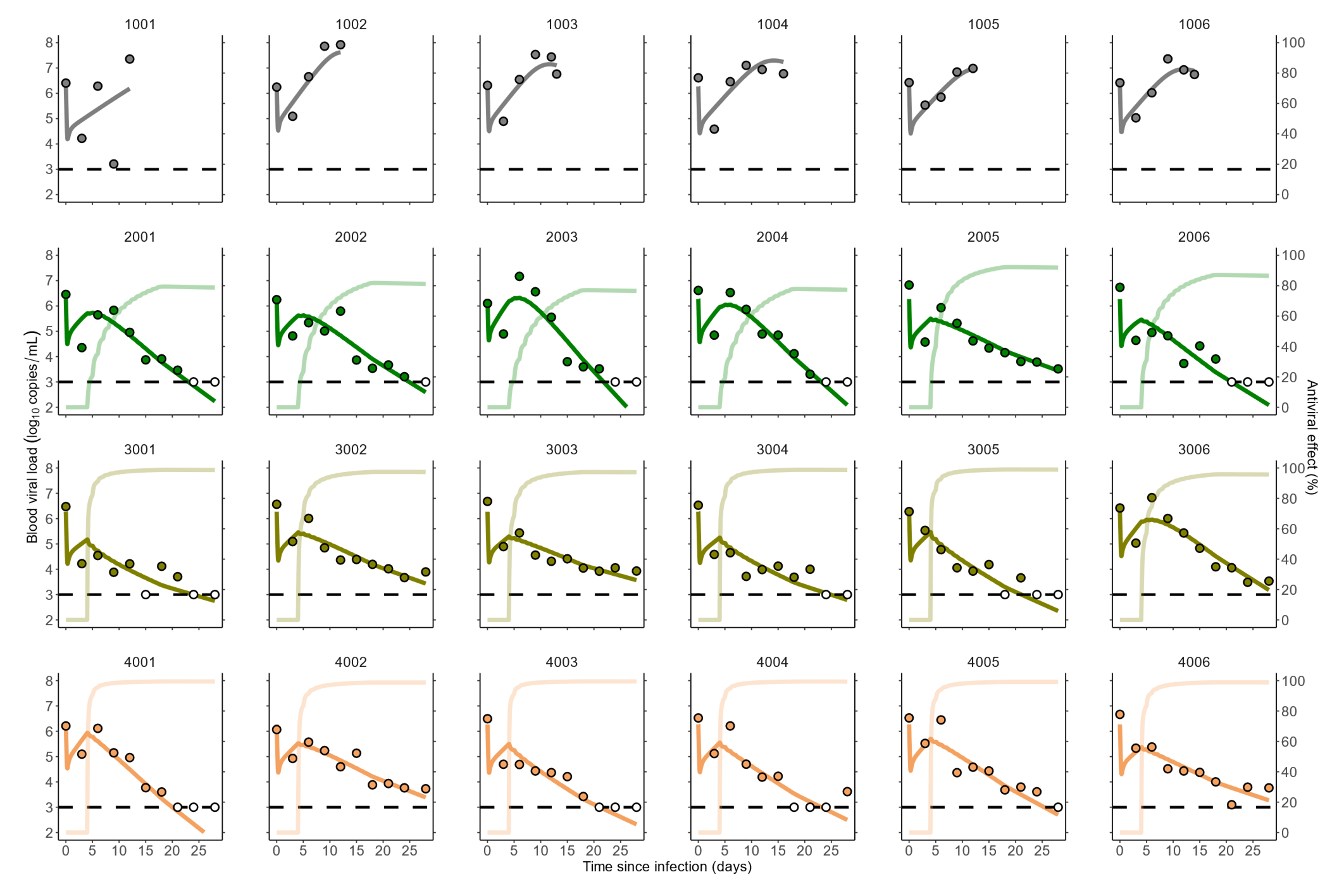

Supplement: S1 Fig — Blood viral load (circles), model predictions for blood viral load (bold solid curves), and for antiviral effect (transparent solid curves). Empty circles indicate data below the limit of quantification by qPCR (LOQ), and horizontal dashed lines indicate the LOQ. Grey: no treatment; green: 3 mg/kg/day; olive: 10 mg/kg/day; orange: 20 mg/kg/day. (TIF) [file pbio.3002249.s002.tif]

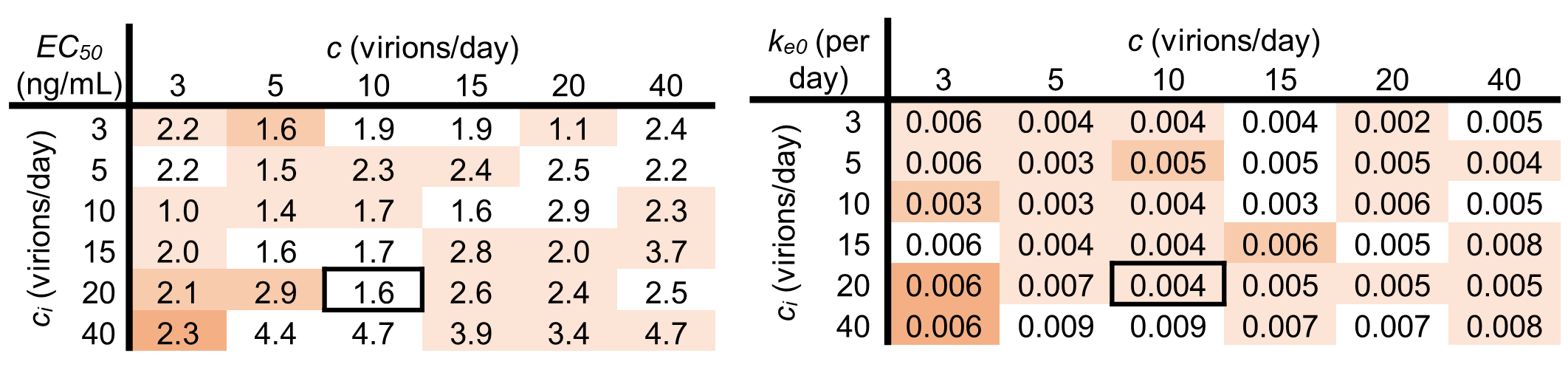

Supplement: S2 Fig — Estimation was performed with different value for each fixed parameter using the final model. Estimated population median values reported for EC50 and ke0. Different colors represent estimates with relative standard error below 50% (white), 50%-100% (light orange), 100%-200% (orange), and over 200% (dark orange). The reference model is c = 10 virions/day and cI = 20 virions/day (black border). (TIF) [file pbio.3002249.s003.tif]

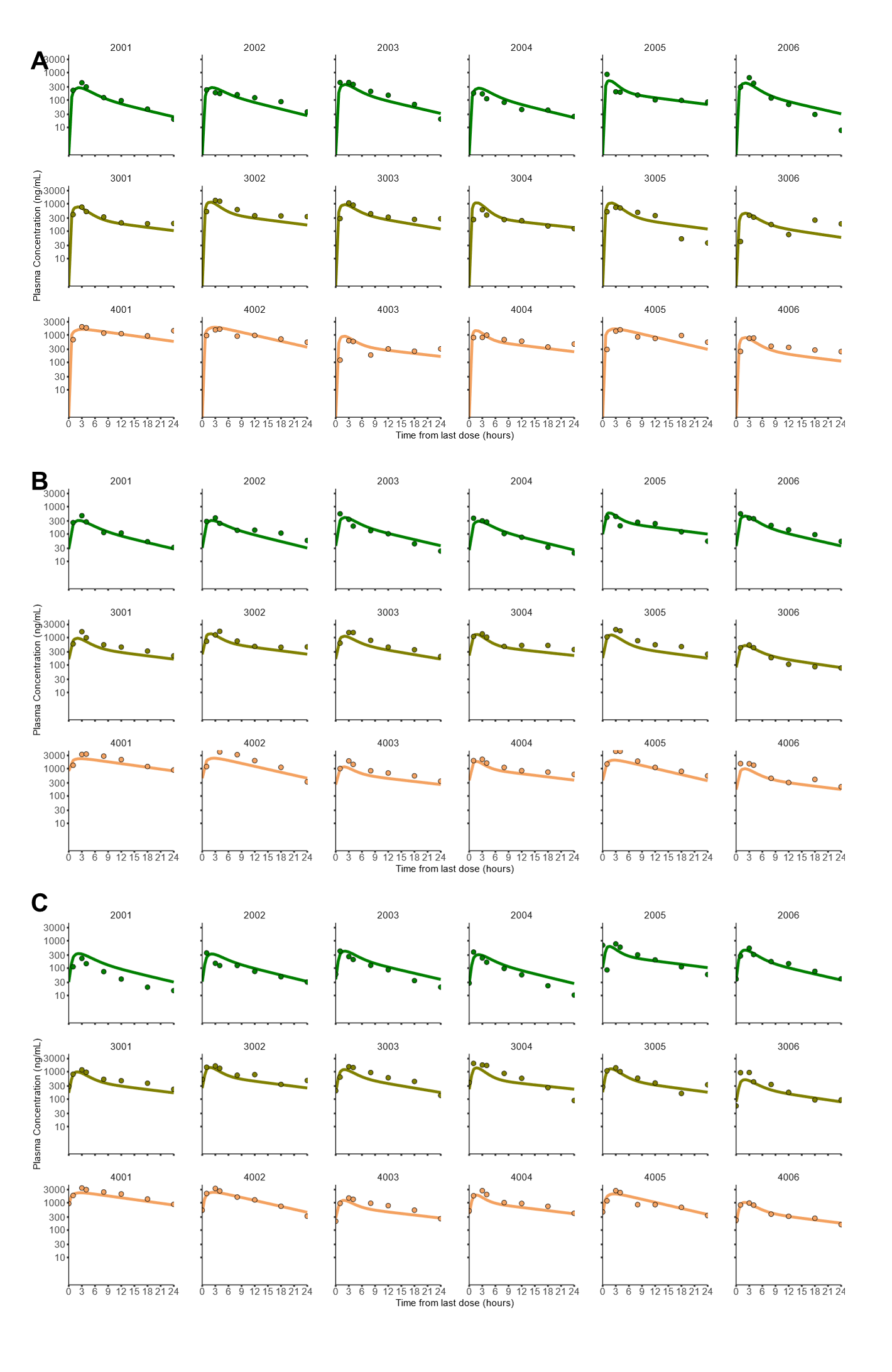

Supplement: S3 Fig — (A) Day 4 postinfection (Day 1 of treatment); (B) Day 10 postinfection (Day 7 of treatment); (C) Day 17 postinfection (Day 14 of treatment). Observed concentrations presented by circles. Model predictions displayed by solid curves. Green: 3 mg/kg/day; olive: 10 mg/kg/day; orange: 20 mg/kg/day. (TIF) [file pbio.3002249.s004.tif]

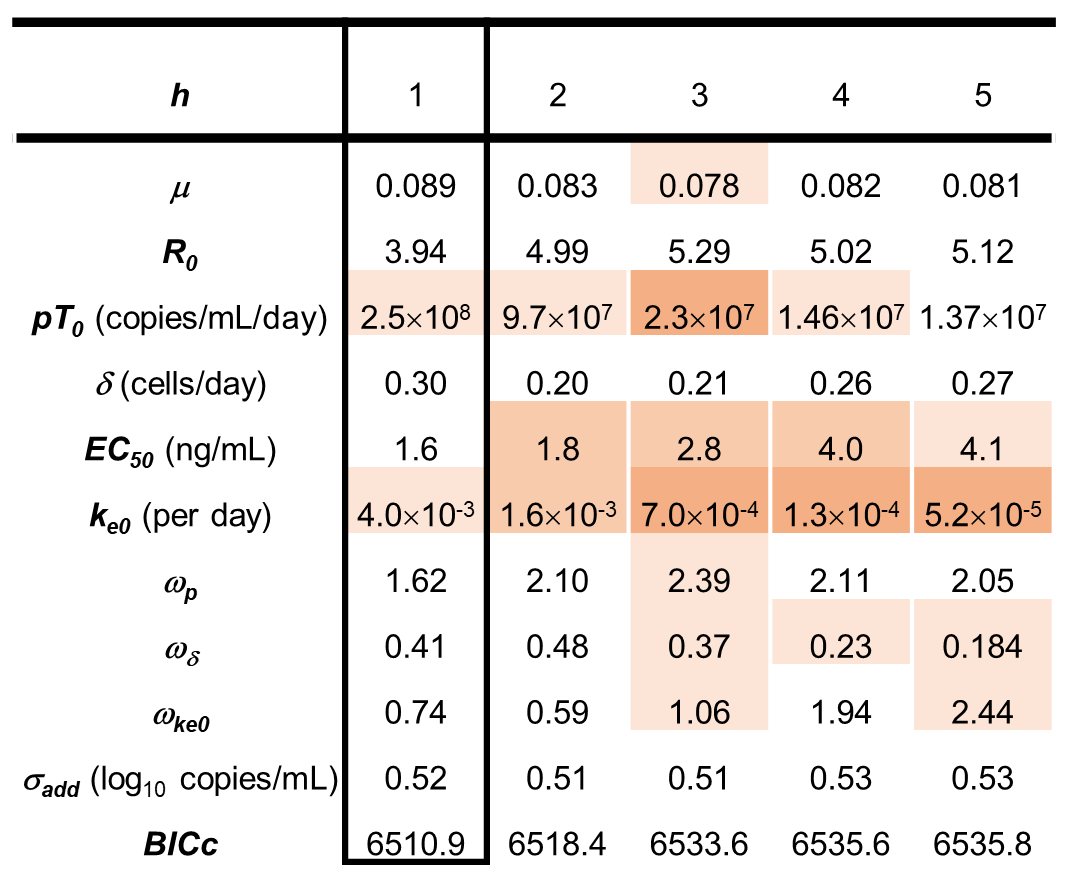

Supplement: S4 Fig — Estimation was performed with different value for each h value. Different colors represent estimates with relative standard error below 50% (white), 50%-100% (light orange), 100%-200% (orange), and over 200% (dark orange). μ, proportion of infectious virions; R0, within-host basic reproduction number; pT0, number of virions produced from infected cells; δ, loss rate of infected cells; EC50, tecovirimat concentrations inhibiting 50% of viral production; h, Hill coefficient in the equation of drug concentration–effect relationship; ke0, drug transfer rate between plasma and effect compartments; ωθ, interindividual variability on parameter θ; σadd, additional error on viral load. The reference model is h = 1 (black border). (TIF) [file pbio.3002249.s005.tif]

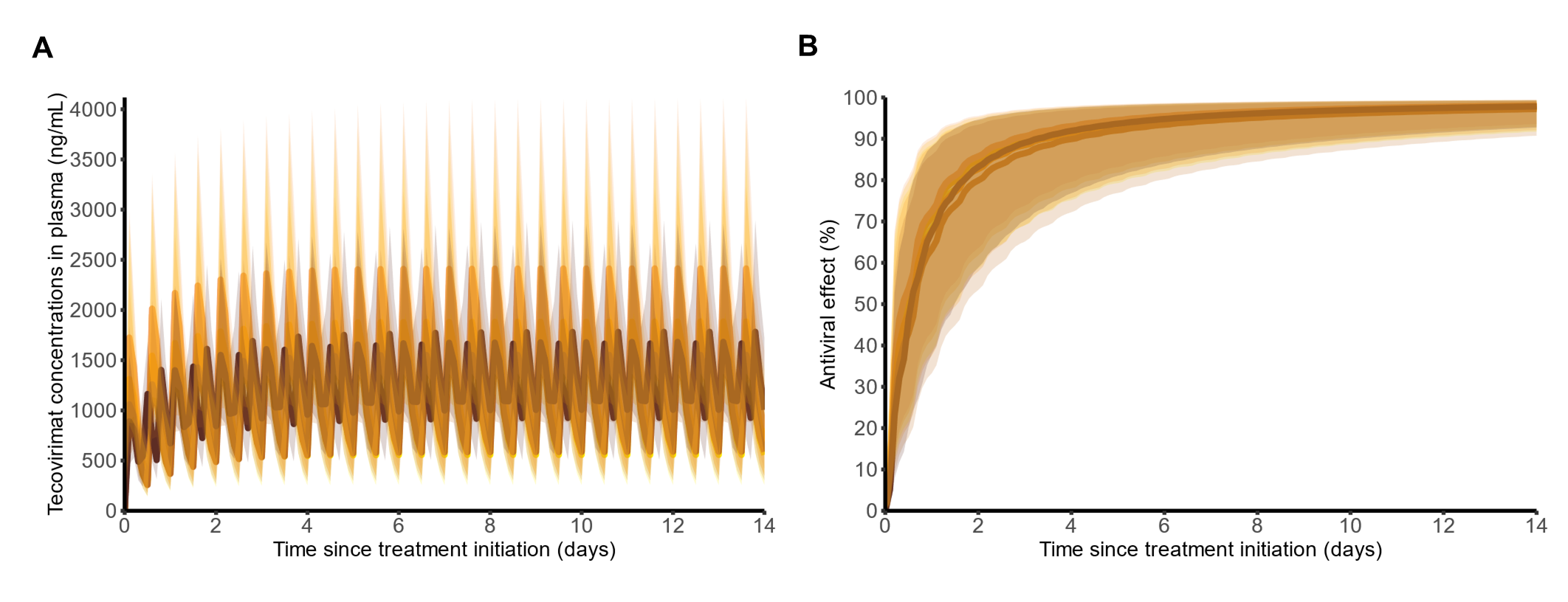

Supplement: S5 Fig — (A) Tecovirimat plasma concentrations over time. (B) Tecovirimat antiviral effect over time. Predictions shown as median and 90% prediction interval, assuming a 14-day treatment course, using pharmacokinetic (PK) parameters estimated in healthy volunteers (S2 Table), and pharmacodynamic (PD) parameters in NHPs (S1 Table). Golden yellow: 200 mg bid for 20 kg; yellow: 400 mg bid for 40 kg; sandy brown: 600 mg bid for 60 kg; dark orange: 600 mg bid for 80 kg; copper: 600 mg bid for 100 kg; brick: 600 mg tid for 120 kg. (TIF) [file pbio.3002249.s006.tif]

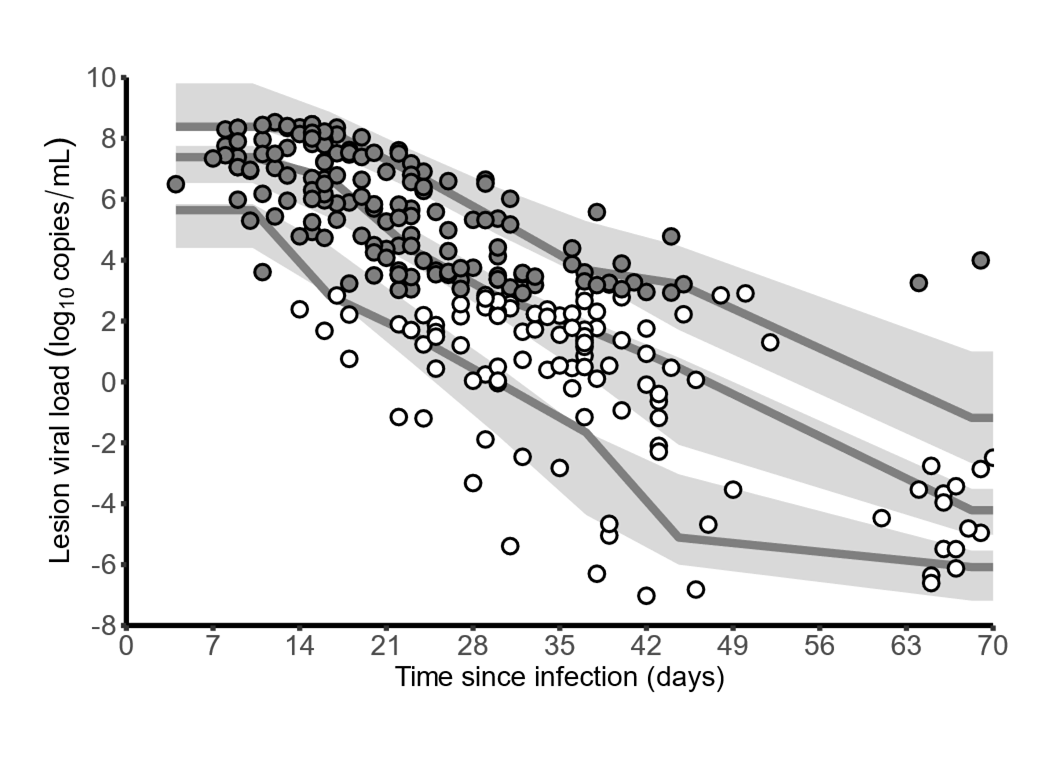

Supplement: S6 Fig — 5th, 50th, and 95th empirical percentiles are displayed in grey lines, and 90% prediction intervals of predicted percentiles in grey ribbons. Observed data are presented by circles, including data below the limit of quantification denoted by white circles. (TIF) [file pbio.3002249.s007.tif]

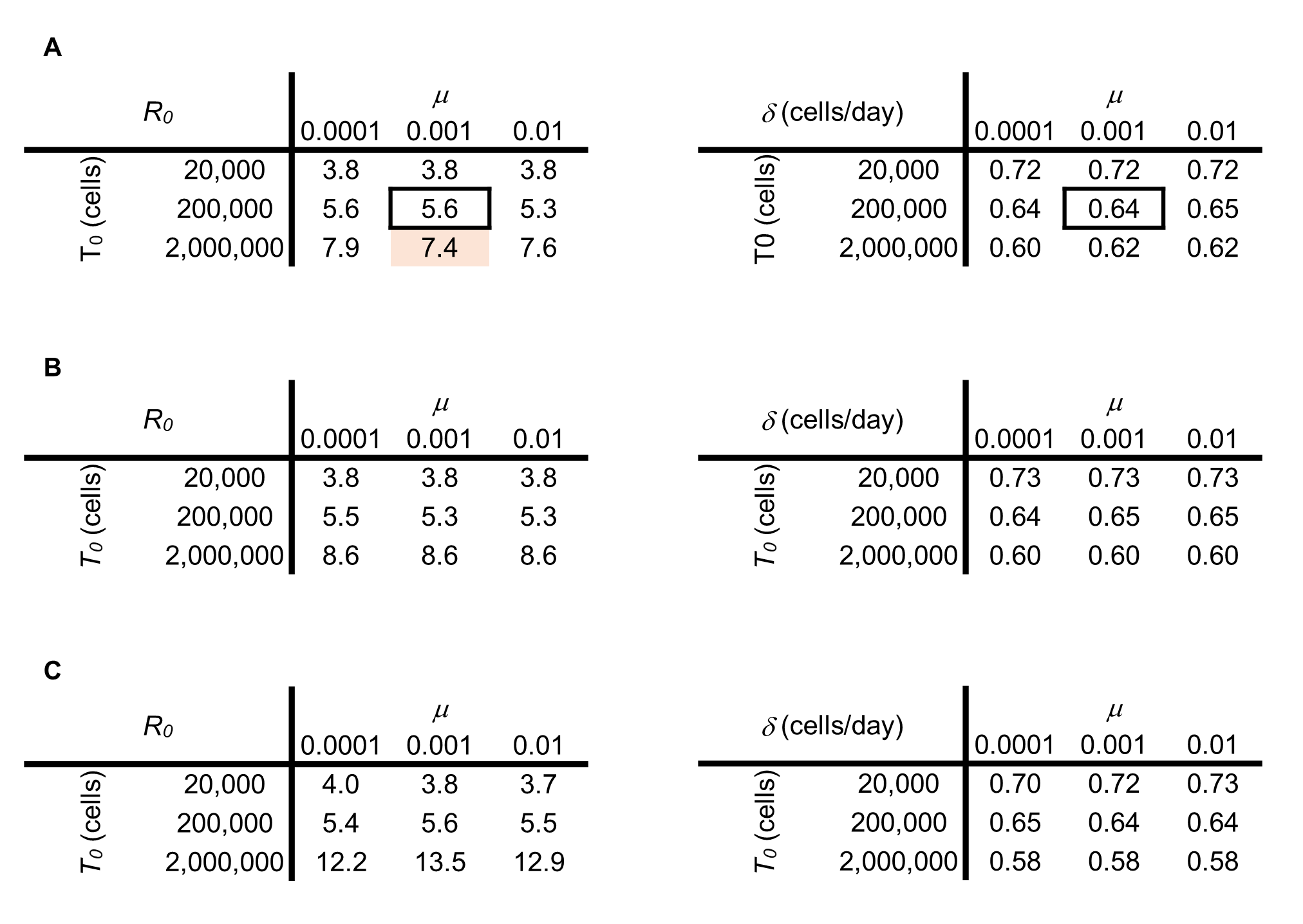

Supplement: S7 Fig — (A) Distribution volume fixed to 0.5 mL. (B) Distribution volume fixed to 0.25 mL. (C) Distribution volume fixed to 0.125 mL. Estimation was performed with different value for each fixed parameter using the final model. Estimated population median values reported for R0 and δ. White and light orange colors represent estimates with relative standard error below 50% and over 50%, respectively. The reference model is μ = 10−3, T0 = 200,000 cells, and the distribution volume of 0.5 mL (black border). (TIF) [file pbio.3002249.s008.tif]

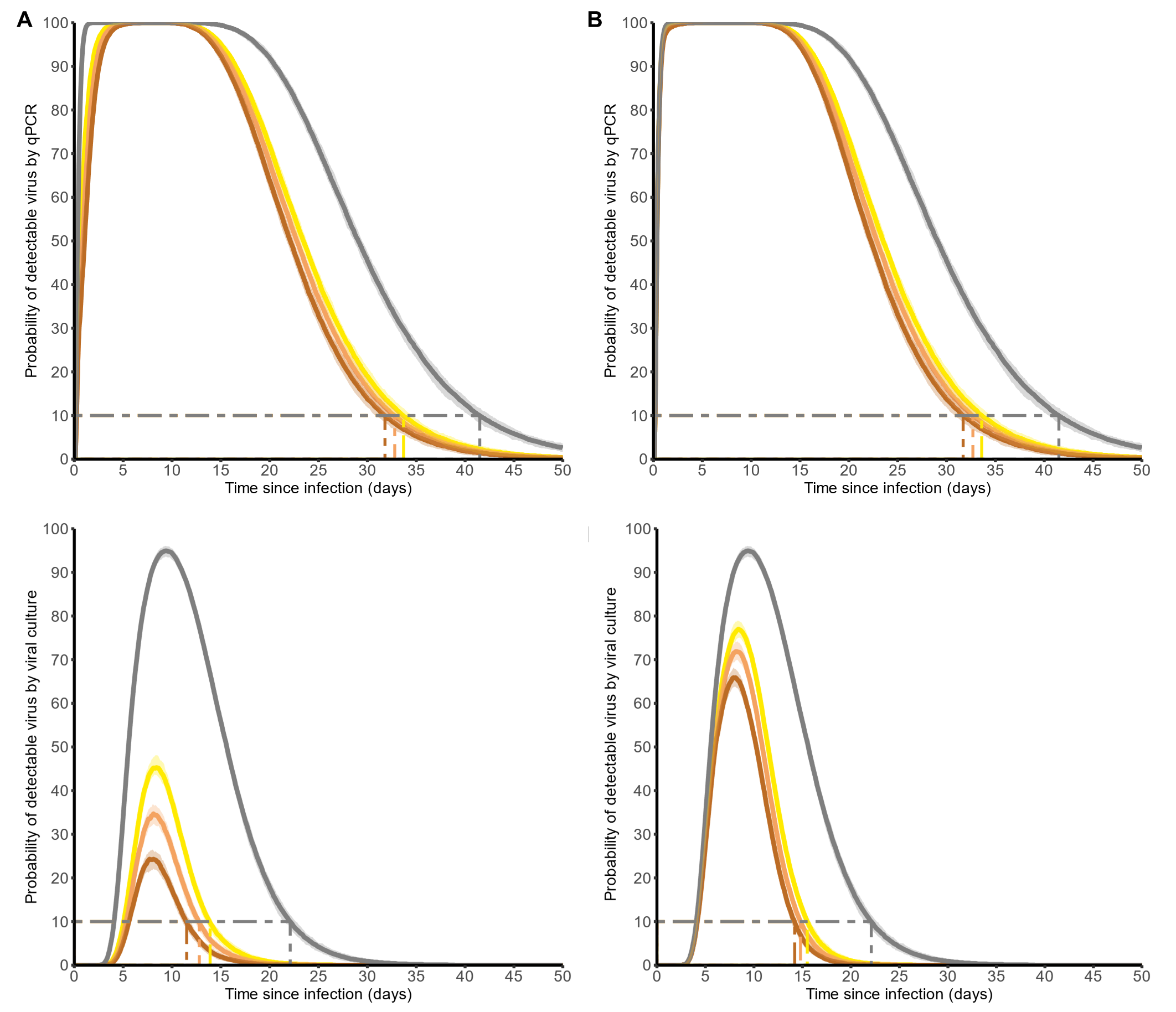

Supplement: S8 Fig — Probability over time of detectable virus by qPCR (A, B), or by viral culture (C, D). Predictions are shown as median and 90% prediction interval, assuming a 14-day treatment course. Limits of quantification (LOQ) by qPCR: 2.9 log10 copies/mL; LOQ by viral culture: 6.5 log10 copies/mL. Grey: no treatment; yellow: 400 mg bid; orange: 600 mg bid; copper: 600 mg tid; dashed lines: time to achieve 90% probability of undetectable virus. (TIF) [file pbio.3002249.s009.tif]

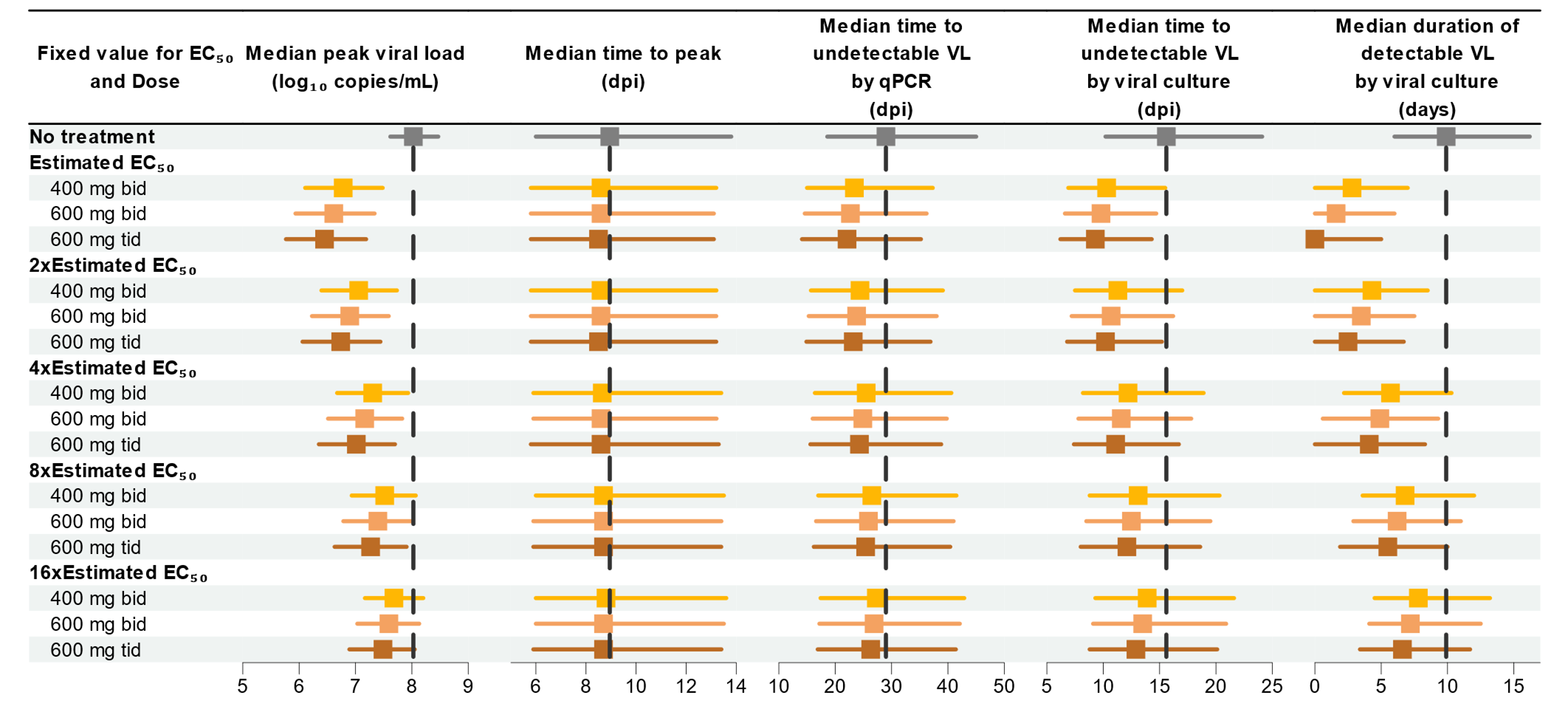

Supplement: S9 Fig — Lesion viral load simulated over time following different postexposure prophylactic doses for each EC50 value. Predictions shown as median and 90% prediction interval, assuming a 14-day treatment course for a 78.4-kg patient, using pharmacokinetic parameters estimated in healthy volunteers (S2 Table), and pharmacodynamic parameters estimated in NHPs (S1 Table); dpi: days postinfection; limit of quantification (LOQ) by qPCR: 2.9 log10 copies/mL; LOQ by viral culture: 6.5 log10 copies/mL. (TIF) [file pbio.3002249.s010.tif]

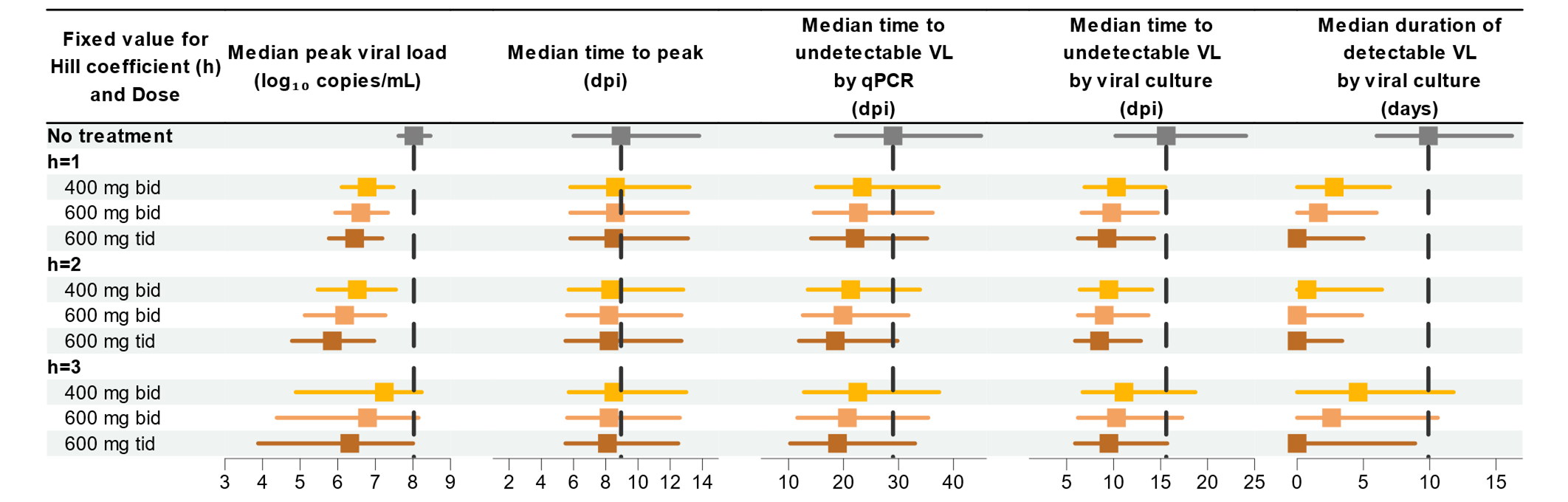

Supplement: S10 Fig — Lesion viral load simulated over time following different postexposure prophylactic doses for each h value. Predictions shown as median and 90% prediction interval, assuming a 14-day treatment course for a 78.4-kg patient, using pharmacokinetic parameters estimated in healthy volunteers (S2 Table), and pharmacodynamic parameters estimated in NHPs (S1 Table); dpi: days postinfection; limit of quantification (LOQ) by qPCR: 2.9 log10 copies/mL; LOQ by viral culture: 6.5 log10 copies/mL. (TIF) [file pbio.3002249.s011.tif]
