# Supplementary material for: Early administration of tecovirimat shortens the time to mpox clearance in a model of human infection
Source: PLoS Biol. 2023 Dec 21;21(12):e3002249. doi: 10.1371/journal.pbio.3002249 (PMC10734935; doi:10.1371/journal.pbio.3002249)
Supplement: S1 Table — PK/PD, pharmacokinetic/pharmacodynamic; NHP, nonhuman primate; RSE, relative standard error; μ, proportion of infectious virions; pT0, number of virions produced from infected cells; δ, loss rate of infected cells; EC50, tecovirimat concentrations inhibiting 50% of viral production; ke0, drug transfer rate between plasma and effect compartments; ωθ, interindividual variability on parameter θ; σadd, additional error on viral load. (DOCX) [file pbio.3002249.s012.docx]

S1 Table. Final estimates for tecovirimat PK/PD model in NHPs (N=24).

| Parameter | Estimate (RSE%) |  |  |
| --- | --- | --- | --- |
| *μ* | 0.09 (24.7) |  |  |
| *R_0_* | 3.94 (16.3) |  |  |
| *pT_0_* (copies/mL/day) | 2.5×10^8^ (95.4) |  |  |
| *δ* (cells/day) | 0.3 (16.2) |  |  |
| *EC_50_* (ng/mL) | 1.6 (43.7) |  |  |
| *k_e0_* (per day) | 4.0×10^-3^ (61.2) |  |  |
| *ω_p_* | 1.62 (38.0) |  |  |
| *ω_δ_* | 0.41 (23.8) |  |  |
| *ω_ke0_* | 0.74 (33.5) |  |  |
| *σ_add_* (log_10_ copies/mL) | 0.52 (6.0) |  |  |
| PK/PD, pharmacokinetic/pharmacodynamic; NHP, non-human primate; RSE, relative standard error; *μ,* proportion of infectious virions; *pT_0_*, number of virions produced from infected cells; *δ*, loss rate of infected cells; *EC_50_*, tecovirimat concentrations inhibiting 50% of viral production; *k_e0_*, drug transfer rate between plasma and effect compartments; *ω_θ_*, inter-individual variability on parameter *θ*; *σ_add_*, additional error on viral load. | | | |
