# Supplementary material for: Early administration of tecovirimat shortens the time to mpox clearance in a model of human infection
Source: PLoS Biol. 2023 Dec 21;21(12):e3002249. doi: 10.1371/journal.pbio.3002249 (PMC10734935; doi:10.1371/journal.pbio.3002249)
Supplement: S2 Table — PK, pharmacokinetic; NHP, nonhuman primates; wt: weight; dose: dose level in mg/kg; IIV, interindividual variability; IOV, intra-occasion variability; NA, not available. (DOCX) [file pbio.3002249.s013.docx]

S2 Table. Final estimates for tecovirimat PK models in mpox-infected NHPs and uninfected humans used in our analysis.

|  | Infected NHP PK parameter | | Uninfected human PK parameter | | |
| --- | --- | --- | --- | --- | --- |
| Parameter | Estimate | IIV (%) | Estimate | IIV (%) |  |
| k_a_ (per hour) | 0.868 ×(dose/10)^0.160^ | 11 | 1.06 | 41 |  |
| T_lag_ (hours) | 0.302 | NA | 1.46 | 17 |  |
| CL/F (liters/hour) | 2.809 × (wt/3.105)^0.75^ × (dose/10)^0.093^ | 31 | 41.15 × (wt/78.4)^0.75^ | 31 |  |
| V_c_/F (liters) | 20.054×(wt/3.105)^1^×(dose/10)^0.623^ | 47 | 217.44 × (wt/78.4)^1^ | 28 |  |
| Q/F (liters/hour) | 3.244 × (wt/3.105)^0.75^ | 75 | 36.79 × (wt/78.4)^0.75^ | 54 |  |
| V_p_/F (liters) | 13.34 × (wt/3.105)^1^ | 55 | 413.53 × (wt/78.4)^1^ | 54 |  |
| Additive error (μg/liter) | 0.133 | NA | 10.92 | NA |  |
| Proportional error (%) | 30 | NA | 27 | NA |  |
| PK, pharmacokinetic; NHP, non-human primates; wt: weight; dose: dose level in mg/kg; IIV, inter-individual variability; IOV, intra-occasion variability; NA, not available. | | | | | |
