# Supplementary material for: Early administration of tecovirimat shortens the time to mpox clearance in a model of human infection
Source: PLoS Biol. 2023 Dec 21;21(12):e3002249. doi: 10.1371/journal.pbio.3002249 (PMC10734935; doi:10.1371/journal.pbio.3002249)
Supplement: S3 Table — Data shown as n (%) or median (IQR); *No participants had a CD4 cell count lower than 100 cells/μL; **Highest count of lesions during the follow-up period. (DOCX) [file pbio.3002249.s014.docx]

S3 Table. Characteristics of patients included in the analyses.

|  | **N = 54** |
| --- | --- |
| Gender |  |
| Men | 53 (98%) |
| Transgender women | 1 (2%) |
| Age, years | 36 (31-46) |
| Coinfection with HIV* | 32 (59%) |
| Recent smallpox vaccination |  |
| Yes | 1 (2%) |
| No | 53 (98%) |
| Incubation period, days | 6.0 (4.0-8.0) |
| Number of localized lesions** |  |
| 0 | 4 (7%) |
| 1 | 11 (20%) |
| 2-5 | 23 (43%) |
| >5 | 16 (30%) |
| Data shown as n (%) or median (IQR);  *No participants had a CD4 cell count lower than 100 cells/μL;  **Highest count of lesions during the follow-up period. | |
