# Supplementary material for: Early administration of tecovirimat shortens the time to mpox clearance in a model of human infection
Source: PLoS Biol. 2023 Dec 21;21(12):e3002249. doi: 10.1371/journal.pbio.3002249 (PMC10734935; doi:10.1371/journal.pbio.3002249)
Supplement: S1 Text — (DOCX) [file pbio.3002249.s015.docx]

S1 Text. The Movie Group

Andrea Alemany, MD^1,2,3^, Eloy José Tarín-Vicente, MD^4^, Adrià Mendoza, MD^1,5^ , Cristina Casañ, PhD^6^ , Vicente Descalzo, MD^7^, Àngel Rivero, MD^2,5^, Pep Coll, MD^2,5^, Xènia Oller, MD^1,2^, José Miguel Cabrera, MD^2,5^, Martí Vall-Mayans, PhD^1,2^, María Dolores Folgueira, PhD^8,9, 10^, Manuel Agud-Dios, MD^4^ , Elena Gil-Cruz, MD^4^, Alexia Paris de Leon^6^, Aída Ramírez Marinero, MD^6^, Vira Buhiichyk^2^, Roger Paredes, PhD^2,11^ , Nuria Prat, MD^12^, Maria-Rosa Sala Farre^13^, Josep Maria Bonet-Simó, MD^12^, Pablo L Ortiz-Romero, PhD^4^, Bonaventura Clotet, PhD^2,11,14,15,16^, Pere-Joan Cardona, PhD^6,17,18^, Ignacio Blanco, PhD^17^

^1^Skin Neglected Diseases and Sexually Transmitted Infections Section, Hospital Universitari Germans Trias i Pujol, Badalona, Spain.

^2^Fight Infectious Diseases Foundation, Badalona, Spain.

^3^Facultat de Medicina, Hospital Clinic, Universitat de Barcelona, Barcelona, Spain

^4^Dermatology Department, Hospital Universitario 12 de Octubre, Madrid, Spain.

^5^BCN Checkpoint, Projecte dels NOMS – Hispanosida, Barcelona, Spain.

^6^Microbiology Department, Clinical Laboratory North Metropolitan Area, University Hospital Germans Trias I Pujol, Badalona, Spain

^7^Infectious Diseases Department, Hospital Universitari Vall d’Hebron, Barcelona, Spain.

^8^Microbiology Department, Hospital Universitario 12 de Octubre, Madrid, Spain.

^9^Instituto de Investigación Sanitaria Hospital 12 de Octubre (imas12), Madrid, Spain.

^10^Department of Medicine, Medical School, Universidad Complutense de Madrid, Madrid, Spain.

^11^Infectious Diseases Department, Hospital Universitari Germans Trias i Pujol, Badalona, Spain.

^12^Direcció d'Atenció Primària - Metropolitana Nord, 08023 Sabadell, Catalonia, Spain.

^13^Public Health Agency of Catalonia, Generalitat de Catalunya, Barcelona, Spain

^14^IrsiCaixa AIDS Research Institute & Fundació Lluita contra les infeccions, Hospital Germans Trias I Pujol, Badalona, Spain.

^15^Universitat de Vic-Universitat Central de Catalunya (UVIC-UCC), Vic, Spain.

^16^Universitat Autònoma de Barcelona (UAB), Barcelona, Spain.

^17^Department of Genetics and Microbiology. Autonomous University of Barcelona, Barcelona, Spain.

^18^Centro de Investigación Biomédica en Red en Enfermedades Respiratorias (CIBERES), Instituto de Salud Carlos III (ISCIII), Madrid, Spain.

| Eloy José | Tarín-Vicente |
| --- | --- |
| Adrià | Mendoza |
| Andrea | Alemany |
| Cristina | Casañ |
| Vicente | Descalzo |
| Àngel | Rivero |
| Pep | Coll |
| Xènia | Oller |
| José Miguel | Cabrera |
| Martí | Vall-Mayans |
| María | Dolores Folgueira |
| Alexia | Paris de Leon |
| Manuel | Agud-Dios |
| Elena | Gil-Cruz |
| Vira | Buhiichyk |
| Roger | Paredes |
| Nuria | Prat |
| Bonaventura | Clotet |
| Pere-Joan | Cardona |
| Ignacio | Blanco |
| Pablo L | Ortiz-Romero |
| Maria-Rosa | Sala Farre |
| Josep Maria | Bonet-Simó |
| Aida | Ramírez Marinero |
