# Supplementary material for: Early administration of tecovirimat shortens the time to mpox clearance in a model of human infection
Source: PLoS Biol. 2023 Dec 21;21(12):e3002249. doi: 10.1371/journal.pbio.3002249 (PMC10734935; doi:10.1371/journal.pbio.3002249)
Supplement: Attachment — Submitted filename: ResponseToReviewers_20231116.docx [file pbio.3002249.s016.docx]

**Early administration of tecovirimat shortens the time to mpox clearance in a model of human infection**

**Bach Tran Nguyen, Aurélien Marc,** **Clara Suñer, Michael Marks, Maria Ubals, Águeda Hernández-Rodríguez, María Ángeles Melendez, The Movie Group, Dennis E. Hruby, Andrew T. Russo, France Mentré, Oriol Mitjà, Douglas W. Grosenbach,** **Jérémie Guedj**

**Response to reviewers**

We thank both anonymous referees and the editor for their insightful comments. All comments have been taken into account in the revised manuscript. Please see below our rebuttal letter.

**Editor’s comments to the author**

Your manuscript has now been evaluated by the PLOS Biology editors, an Academic Editor with relevant expertise, and by several independent reviewers. In light of the reviews, which you will find at the end of this email, we are pleased to offer you the opportunity to address the comments from the reviewers in a revision that we anticipate should not take you very long. We will then assess your revised manuscript and your response to the reviewers' comments with our Academic Editor aiming to avoid further rounds of peer-review, although might need to consult with the reviewers, depending on the nature of the revisions. Please also address the following policy and editorial issues:

1. We suggest a change in the title along these lines: "Early administration of tecovirimat shortens the time to mpox clearance in a model of human infection".

We thank you and the Reviewers for your time and very valuable feedback. We have made substantial revisions to the manuscript and believe it is now a much stronger paper.

We fully agree with your suggestion and modified the title accordingly.

2. Please describe all sources of funding that have supported your work. This information is required for submission and will be published with your article, should it be accepted. A complete funding statement should do the following:

- Include grant numbers and the URLs of any funder's website. Use the full name, not acronyms, of funding institutions, and use initials to identify authors who received the funding.

- Describe the role of any sponsors or funders in the study design, data collection and analysis, decision to publish, or preparation of the manuscript. If the funders had no role in any of the above, include this sentence at the end of your statement: "The funders had no role in study design, data collection and analysis, decision to publish, or preparation of the manuscript.

Required information was included in the revised manuscript accordingly.

3. Please provide a blurb which (if accepted) will be included in our weekly and monthly Electronic Table of Contents, sent out to readers of PLOS Biology, and may be used to promote your article in social media. The blurb should be about 30-40 words long and is subject to editorial changes. It should, without exaggeration, entice people to read your manuscript. It should not be redundant with the title and should not contain acronyms or abbreviations.

We thank the Editors for this suggestion. Please find the blurb to be included in social media as follows:

*“The impact of early tecovirimat administration on the time to viral clearance in patients with mpox infection is predicted using an integrative modeling approach combining pre-clinical and clinical data.”*

4. ETHICS STATEMENT:

-- Please include the full name of the IACUC/ethics committee that reviewed and approved the animal care and use protocol/permit/project license. Please also include an approval number.

-- Please include the specific national or international regulations/guidelines to which your animal care and use protocol adhered. Please note that institutional or accreditation organization guidelines (such as AAALAC) do not meet this requirement.

-- Please include information about the form of consent (written/oral) given for research involving human participants. All research involving human participants must have been approved by the authors' Institutional Review Board (IRB) or an equivalent committee, and all clinical investigation must have been conducted according to the principles expressed in the Declaration of Helsinki.

All required information was included in the revised manuscript as follows:

“*The NHP study was conducted in compliance with U.S. FDA 21 CFR Part 58 (Good Laboratory Practice for Nonclinical Laboratory Studies). Exceptions to U.S. FDA 21 CFR Part 58 include the manufacturing and analysis of the test article, which will be performed under GMP, and characterization of the test pathogen (MPXV). The protocol also complied with all applicable sections of the Final Rules of the Animal Welfare Act regulations (9 CFR Parts 1, 2, and 3) and Guide for the Care and Use of Laboratory Animals - National Academy Press, Washington D.C. 1996 (the Guide). This study was conducted in LBERI's (Lovelace Biomedical and Environmental Research Institute, Albuquerque, New Mexico) AAALAC (Association for the Assessment and Accreditation of Laboratory Animal Care)-accredited facility. The study was supported by the Division of Microbiology and Infectious Diseases, National Institute of Allergy and Infectious Diseases, National Institutes of Health (contract HHSN266200600014C), and the Biomedical Advanced Research and Development Authority (contract HHSO100201100001C).*

*The study protocol of human viral dynamics [7] was approved by the Ethics Committee of the Hospital Germans Trias i Pujol (PI-22-156) and written informed consent was obtained from all participants before enrolment. All investigation were conducted according to the principles expressed in the Declaration of Helsinki.*”

5. DATA POLICY: You may be aware of the PLOS Data Policy, which requires that all data be made available without restriction: http://journals.plos.org/plosbiology/s/data-availability. For more information, please also see this editorial: <http://dx.doi.org/10.1371/journal.pbio.1001797>

Note that we do not require all raw data. Rather, we ask that all individual quantitative observations that underlie the data summarized in the figures and results of your paper be made available in one of the following forms:

A) Supplementary files (e.g., excel). Please ensure that all data files are uploaded as 'Supporting Information' and are invariably referred to (in the manuscript, figure legends, and the Description field when uploading your files) using the following format verbatim: S1 Data, S2 Data, etc. Multiple panels of a single or even several figures can be included as multiple sheets in one excel file that is saved using exactly the following convention: S1_Data.xlsx (using an underscore).

B) Deposition in a publicly available repository. Please also provide the accession code or a reviewer link so that we may view your data before publication.

Regardless of the method selected, please ensure that you provide the individual numerical values that underlie the summary data displayed in the following figure panels as they are essential for readers to assess your analysis and to reproduce it: Figures 1CD, 2AB, 3ABC, 4, and Supplementary Figures S1, S3ABC, S4AB, S5, S7ABCD.

NOTE: the numerical data provided should include all replicates AND the way in which the plotted mean and errors were derived (it should not present only the mean/average values).

**Please also ensure that figure legends in your manuscript include information on where the underlying data can be found, and ensure your supplemental data file/s has a legend.**

Please ensure that your Data Statement in the submission system accurately describes where your data can be found.

We thank the Editors for this information. Required data was included for each figure in the recommended formatting.

REVIEWS:

Reviewer #1: This is a great paper that is clearly written and captures the benefit of mathematical modeling for clinical trial simulation. This is really the perfect use of modeling to inform future experiments and trials. Consideration of viral dynamics in addition to PK/PD models adds another critical dimension toward optimizing the likelihood of therapeutic efficacy in clinical trials. I only have minor points:

1) The authors point out that the EC50 derived based on reducing viremia in non-human primates may not be equivalent to that required for reducing viral load in skin lesions in humans. Estimates for other antivirals have suggested higher concentrations needed in vivo relative to in vitro. A sensitivity analysis in which the human *in vivo* EC50 is higher would provide a more conservative approximation of in vivo efficacy and would make a nice supplementary figure. It would be interesting to see if doses in humans start to matter more if the actual in vivo EC50 in humans is higher than that in non-human primates.

We greatly appreciate the Reviewer’s comments and helpful suggestions. A sensitivity analysis with higher *EC_50_* than our final estimation was added to the article as a supplementary figure (S9 Fig). Our analysis shows that the results remain largely unchanged for a human *EC_50_* up to 10-fold higher than estimated in non-human primates.

2) The mechanism of action of tecovirimat should be mentioned somewhere in the paper to justify that it impacts viral replication rather than infectivity / viral entry (beta).

We thank the Reviewer for this comment. Actually, the mechanism of action was mentioned in Materials and Methods Section to justify how our model predicts the antiviral effect. However, we agree that it would be better to add more data on tecovirimat pharmacology, including the mechanism of action. These were included into the Introduction Section as below:

“[…] *Initially developed to treat human smallpox, tecovirimat demonstrated strong antiviral activity by blocking the VP37 protein in virus maturation and release from the infected cell [8,9]. The drug pharmacokinetics (PK) and tolerance were assessed in animals and healthy volunteers to propose relevant dosing regimens [9,10], showing an oral bioavailability of 48%, higher with food intake, and high plasma protein binding (75-82%). Tecovirimat is metabolized mostly to 4-trifluoromethyl benzoic acid (TFMBA) with no pharmacologically active metabolites [11].”*

3) Was the Hill coefficient considered or estimated in vitro? Could this impact viral load trajectories if varied?

We thank the Reviewer for bringing up this point. The model assumes that tecovirimat inhibit viral production through an effect compartment, which leads to smooth the antiviral efficacy as compared to a direct effect model. To ensure parameter identifiability, we fixed h=1. A sensitivity analysis has now been added, assuming h=2 to 5. These values lead to higher *EC_50_* and lower *k_e0_*, but did not improve precision in the estimation or Bayesian Information Criteria (S4 Fig - Fig 1 shown below). Finally, simulations using higher Hill coefficients (h=2-5) showed no major impact on viral kinetics (S10 Fig - Fig 2 shown below).

**Figure 1. Sensitivity analysis on model estimates with different Hill coefficient value.**

Estimation was performed with different value for each fixed h value. Different colors represent estimates with relative standard error below 50% (white), 50-100% (light orange), 100-200% (orange), and over 200% (dark orange). *μ*, proportion of infectious virions; *R_0_*, within-host basic reproductive ratio; *pT_0_*: number of virions produced from infected cells; *δ*, loss rate of infected cells; *EC_50_*, tecovirimat concentrations inhibiting 50% of viral production; *h*, Hill coefficient in the equation of drug concentration-effect relationship; *k_e0_*, drug transfer rate between plasma and effect compartments; *ω_θ_*, inter-individual variability on parameter *θ*; *σ_add_*, additional error on viral load. The reference model is *h*=1 (black border).

**Figure 2. Sensitivity analysis on tecovirimat effect in mpox-infected humans with different Hill coefficient value.**

Tecovirimat antiviral effect (A, C, E) and lesion viral load (B, D, F) over time following different prophylactic doses. For each fixed value of Hill coefficient: *h*=1 (A, B), *h*=2 (C, D) and *h*=3 (E, F), predictions shown as median and 90% prediction interval, assuming a 14-day treatment course for a 78.4-kg patient, using pharmacokinetic parameters estimated in healthy volunteers (Supplementary Table 2), and pharmacodynamic parameters estimated in non-human primates (Table 1). Grey: no treatment; yellow: 400 mg bid; orange: 600 mg bid; copper: 600 mg tid.

4) This is picky but target cell limitation seems like the wrong term for this infection. Clearly there is not target cell limitation for human mpox lesions as most genital skin is left uninfected even during severe infections. This is easily visible to the naked eye. While the model implies target cell limitation, I think it would be good to acknowledge that what may really be occurring is a combination of spatial constraint on virus spread as well as innate immune effects. It is fine these details are not captured with the model but the over simplification of the target cell limitation assumption should be noted.

We completely agree with the Reviewer and added this limitation to the Discussion Section:

*“This model also assumes that viral clearance is mediated by target cell limitation, which is a very strong assumption. This might be incorrect for human mpox lesions as most of genital skin is left uninfected, even during severe infections. Our model might oversimplify how spatial constraint and innate immune effects impact virus spread.”*

5) Equating culture positivity with infectivity is an oversimplification as no formal surrogate for transmissibility has been established for this virus. I might say that culture is likely to be a more specific marker of infectiousness than PCR.

The Reviewer is quite correct. The term “duration of infectiousness” has been removed throughout the paper and replaced by “duration of positive viral culture”. A paragraph was added to the Discussion, implying that culture positivity may be a more specific proxy of infectiousness:

*“Tecovirimat may reduce viral replication from infected cells by more than 99% in humans, and could shorten the time to viral clearance and the duration of positive viral culture, provided that treatment is initiated before viral peak (which occurs about two days after symptom onset), or even earlier, ideally as an immediate post-exposure prophylaxis.* *This suggests potential benefits in shortening infectious periods following early administration of tecovirimat, as viral culture* *positivity may be a better proxy of infectiousness than genomic RNA [7].”*

Reviewer #2: This is an outstanding manuscript that describes a modeling approach to analyze mpox kinetics under tecovirimat treatment in humans. The authors pool data from non-human primates, healthy volunteers and a small group of patients to develop a kinetic model of expected treatment effects in humans. I can only congratulate the authors on this work. The manuscript addresses a highly relevant topic, and given small patient numbers, the approach taken by the authors is an excellent way to gain information about expected treatment effects and optimize treatment schedules, here leading to the recommendation to use tecovirimat immediately after exposure. The manuscript is clearly written, and the models and methods employed are sound. The results are convincing. I have - and this is an absolute exception - no further requests for changes and find the paper ready for publication in its present form.

It is absolutely a great honor to find your appreciation to our work. We are highly grateful for your positive and helpful evaluation.

Reviewer #3: This is an interesting bridging study to link pharmacokinetic-pharmacodynamic (PK-PD) relationships for tecovirimat in human mpox disease. The manuscript is clearly written and the topic is important for the field. There are some points that would help clarify the methods and findings for readers.

1. There was a difference in macaque PK for mpox infected versus uninfected animals (conc were lower in infected animals). Was this considered for bridging the PK to humans (i.e., that mpox infected humans could have lower concentrations compared with healthy volunteers upon which the PK for this study was based?). If not, this should be listed as a limitation.

We agree with the Reviewer that mpox infection may possibly impact the PK in humans as described in NHPs. Unfortunately, lacking data reported in patients treated with tecovirimat prevents us from formally assessing this aspect. We therefore added this into the Discussion section as a limitation:

*“Bridging data from NHPs to humans is contingent upon several hypotheses. First, the experimental model of mpox infection in NHPs is more severe than in humans, with an extensive and prolonged viremia in all animals, compared to the transient and low-level viremia generally observed in humans [4,6,7]. Additionally, the NHP model suggested that mpox infection could modify tecovirimat PK, requiring drug exposure analysis in clinical studies [10,14].”*

2. I agree with the basic conclusions from the paper - that the model supports clinically relevant tecovirimat activity for mpox in humans, but it is based on a model that bridges macaque and human data to make estimates. There are many necessary assumptions and the authors address some, but not ones that are unforeseen. I think the conclusions/discussion should acknowledge that unforeseen assumptions could change the model outcomes and the conclusions. Also, the authors should go through the paper and make sure the model findings are referred to as "estimates" or "model predictions" throughout.

We have included the Reviewer’s comment in the revised manuscript. All model findings were checked to be referred as “estimates” or “predictions”.

3. Please define the omegas and sigma in table 1.

We highly appreciate the Reviewer for this comment. This table shows all parameters used to simulate tecovirimat effect on viral kinetics in human skin lesions. All definitions were included into the revised manuscript as suggested.

4. Please include a few sentences on key pharmacologic properties of tecovirimat: mechanism of action, plasma protein binding, bioavailability, metabolic fate.

We thank the Reviewer for this suggestion. Data on tecovirimat pharmacology were added to the Introduction:

*“Initially developed to treat human smallpox, tecovirimat demonstrated strong antiviral activity by blocking the VP37 protein in virus maturation and release from the infected cell [8,9]. The drug pharmacokinetics (PK) and tolerance were assessed in animals and healthy volunteers to propose relevant dosing regimens [9,10], showing an oral bioavailability of 48%, higher with food intake, and high plasma protein binding (75-82%). Tecovirimat is metabolized mostly to 4-trifluoromethyl benzoic acid (TFMBA) with no pharmacologically active metabolites [11].”*

5. The authors mention dose dependent PK but the human models do not include dose on clearance or volume. Was this just in the macaque model?

We confirm that dose-dependent PK of tecovirimat was only found in macaques and not in humans. More details can be found in the reference article number 10 and 14.

6. The colors in the figure (yellows and oranges) are difficult to distinguish. consider more contrasting colors, or dashes, etc. to help reader distinguish the different curves/groups.

We totally agree with the Reviewer about this helpful suggestion. Colors were modified to well distinguish different treated groups.

7. The k_e0_ (effect site rate constant) generates a concentration in an effect compartment, but this appears to be completely arbitrary as the authors have no pharmacologic data in such a compartment with which to verify this part of the model. this is relevant for the *EC_50_* which appears to be from the effect site compartment concentration, thus not verifiable. Is it possible to report an *EC_50_* based upon plasma concentrations? This reviewer understands the importance of a lag time to reach the effect compartment concentration, but a plasma *EC_50_* (if available from the model) is much more relevant for the field.

Ideally, a plasma PK target would be highly relevant in the perspective of therapeutic monitoring. Given that the critical efficacy is estimated to 82%, the relevant PK marker should be EC_80_ or EC_90_, rather than EC_50_. To assess whether appropriate exposure in the effect compartment can be inferred from plasma drug concentrations, we reevaluated our simulations by analyzing tecovirimat trough concentrations (*C_min_*) in plasma according to the predicted pharmacodynamic response (i.e., antiviral efficacy level in the effect compartment, *ε*). Unfortunately, our model could not show any differences in tecovirimat plasma concentrations between individuals with adequate antiviral efficacy and those remaining below the critical threshold (see Figures below). These results will need revisiting in future PK/PD clinical studies. This was added in the discussion as follows:

*“**[…] The EC_50_ was reported based on an effect compartment, lacking pharmacological data to verify this part of the model. Consequently, it might be* *more clinically beneficial to propose plasma concentrations as a PK/PD target. To evaluate whether plasma concentrations are associated with antiviral efficacy in the effect compartment, using our model we analyzed simulated plasma concentrations by groups of adequate antiviral efficacy and those remaining below the critical threshold. However, our model could not identify any differences in plasma drug concentrations between these groups (not shown). This prediction will need revisiting with upcoming PK/PD clinical results.”*

**Figure 3. Plasma concentrations (left) and antiviral effect (*ε*, right) following tecovirimat 600 mg bid.**

**Figure 4. Plasma trough concentrations by groups of antiviral effect (*ε*) on Day 2 (left) and Day 8 (right) after initiating tecovirimat 600 mg bid.**
